# Highway Crossing Rates of Wild Felids Before, During, and After Wildlife Crossing Structure Installation

**DOI:** 10.1002/ece3.70703

**Published:** 2024-12-23

**Authors:** Sarah E. Lehnen, Mitch A. Sternberg, Hilary M. Swarts, John H. Young, Victoria Hanley, Richard J. Kline

**Affiliations:** ^1^ U.S. Fish and Wildlife Service Albuquerque New Mexico USA; ^2^ South Texas Refuge Complex United States Fish and Wildlife Service Alamo Texas USA; ^3^ Laguna Atascosa National Wildlife Refuge United States Fish and Wildlife Service Los Fresnos Texas USA; ^4^ Environmental Affairs Division Texas Department of Transportation Austin Texas USA; ^5^ School of Earth, Environmental and Marine Sciences University of Texas Rio Grande Valley Brownsville Texas USA

**Keywords:** bobcat, *Leopardus pardalis*, *Lynx rufus*, ocelot, wildlife crossing structure, wildlife–vehicle collisions

## Abstract

In an environment increasingly dominated by roads, wildlife crossing structures (WCS) have been installed to decrease wildlife mortality and improve habitat linkages. In South Texas, vehicle collisions have been a major mortality source for the endangered ocelot ( *Leopardus pardalis* ). To mitigate threats to this species, eight WCS, along with associated fencing, were strategically placed along Farm‐to‐Market Road 106 (FM106), which passes through ocelot habitat. We assessed the rate of on‐roadway crossings and WCS use by GPS‐collared animals before, during, and after WCS installation using camera traps to verify WCS use. Because of the rarity of ocelots in this region, we supplemented ocelot data with data from bobcats ( *Lynx rufus* ), a similarly sized felid with slightly more general habitat preferences. Thirty‐five animals, 30 bobcats, and 5 ocelots were tracked using GPS collars between 2013 and 2021. We observed a decline in the total number of crossings and on‐roadway crossings of FM106 for both ocelots and bobcats over the duration of the study, despite controlling for factors such as an individual animal's proximity to FM106. There were 37 documented crossings using WCS by GPS‐marked bobcats while their collars were active. However, the rate of on‐roadway crossings during the post‐construction period was still higher than the WCS use rate for both species. Animals were more likely to make on‐roadway crossings during nighttime especially during unusually bright nights. While controlling for other factors, both species moved more quickly when making on‐roadway crossings compared to WCS crossings or noncrossing intervals but moved more slowly when traffic levels were higher than expected for that time of day. Animals may take years to acclimate to the presence of WCS, suggesting that the use of these structures may increase over time. Additional fencing could further help limit high‐risk road encounters by ocelots in this region.

## Graphical abstract

In South Texas, eight wildlife crossing structures (WCS) were installed to mitigate vehicle collisions and improve habitat connectivity for the endangered ocelot and other wildlife. GPS tracking of 35 animals (30 bobcats and 5 ocelots) from 2013 to 2021 showed a decline in total and on‐roadway crossings, and, although WCS were used, they were still used less frequently than on‐roadway crossings. The study suggests that animals may need time to acclimate to WCS, and additional fencing could further reduce high‐risk road encounters.

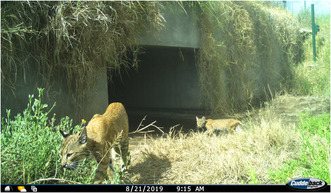

## 1 Introduction

Roads have generally negative effects on surrounding wildlife through vehicle collisions, barriers to movement, habitat loss and fragmentation, and degradation of habitat (Taylor and Goldingay 2010; Rytwinski and Fahrig 2015; van der Ree, Smith, and Grilo 2015). Vehicle collisions are among the most direct impacts of roads and have been observed to be responsible for 30%–40% of all mortalities in some wildlife populations (Jaeger and Fahrig 2004). A literature review on the impacts on mammals by roads found that in 28% of the studies, wildlife–vehicle collisions were the highest cause of mortalities (Moore et al. 2023). In addition, the global impacts of roads are likely to increase, with an expected increase of 14%–23% in road length by 2050 (Meijer et al. 2018). Given the magnitude and projected increase in the threat of roads to wildlife, mitigating these effects is a pressing contemporary conservation issue (Ibisch et al. 2016; Barrientos et al. 2021).

Felids have garnered conservation interest concerning road mitigation because urbanization and roads have been linked to reduced habitat suitability and increased mortality risk for these species (Dickson and Beier 2002; Burdett et al. 2010; Poessel et al. 2014; Schmidt, Lewison, and Swarts 2020; Blackburn, Heffelfinger et al. 2021). In the United States, the ocelot ( *Leopardus pardalis* ) is critically endangered, with breeding populations found only in South Texas (United States Fish and Wildlife Service (USFWS) 2016). The population persistence of ocelots in South Texas is threatened by road‐impacted and fragmented habitats (Haines, Tewes, and Laack 2005; Janečka et al. 2014; Schmidt, Lewison, and Swarts 2020; Blackburn, Heffelfinger et al. 2021). In South Texas, vehicle collisions have been identified as the leading cause of known deaths among ocelots, contributing to at least 40% of the mortality events (Blackburn, Heffelfinger et al. 2021; Haines, Tewes, and Laack 2005; Schmidt, Lewison, and Swarts 2020).

Bobcats ( *Lynx rufus* ) are also present in South Texas and are considered more of a generalist species tolerant to some degree of urbanization (Riley, Gehrt, and Cypher 2010; Young et al. 2019; Lombardi, MacKenzie et al. 2020) that select for areas with less vegetation than ocelots (Horne et al. 2009; Sergeyev, Cherry et al. 2023). However, Schmidt, Lewison, and Swarts (2020) found bobcats to be a reasonable surrogate for ocelots in terms of habitat use. In addition, despite bobcat tolerance of habitat fragmentation, researchers have suggested that maintaining corridors for travel between habitat fragments is necessary for bobcat population persistence (Tigas, Van Vuren, and Sauvajot 2002; Litvaitis et al. 2015), suggesting that this species would also benefit from greater connectivity. Farm‐to‐Market Road 106 (FM106) is a major roadway that bisects ocelot habitat within Laguna Atascosa National Wildlife Refuge (LANWR) in South Texas, a region that hosts one of the two remaining ocelot populations in the United States (USFWS 2016). Increases in human population and road networks in this region have heightened the risk of vehicle collisions to wild felids in this region and this risk is expected to continue to increase (Lombardi, Perotto‐Baldivieso, and Tewes 2020).

To reduce the harmful effects of roads on wildlife, especially on endangered or protected species, transportation agencies in coordination with resource managers sometimes install wildlife crossing structures (WCS) to enable animals to safely traverse roads. Fencing may be used in conjunction with WCS, to funnel wildlife toward WCS, increasing the likelihood individuals will use the WCS and decreasing the chance they will attempt to cross by moving across the roadway (McCollister and Van Manen 2010). In response to the dangers presented by this road, the United States Fish and Wildlife Service (USFWS) and the Texas Department of Transportation (TxDOT) partnered to install WCS at locations where ocelots have been documented crossing or where USFWS biologists believed they were likely to cross, based on the presence of dense closed canopy habitat, the preferred habitat of ocelots in South Texas (Laack 1991; Shindle and Tewes 1998; Harveson et al. 2004), adjacent to the road (Blackburn, Anderson et al. 2021).

The objectives of this study were to identify the effectiveness of WCS in reducing risky on‐roadway crossings by bobcats and ocelots by assessing movement before, during, and after installation of eight under‐the‐road, culvert‐style WCS. We examined on‐roadway crossing rates and total crossing rates over time to evaluate how the WCS affected the rate of on‐roadway risk events and habitat connectivity. In addition, we assessed how speed of travel was affected by crossing events (on‐roadway or use of WCS) and assessed the timing and conditions when each type of crossing event was most likely to occur.

## 2 Materials and Methods

### 2.1 Study Area

**FIGURE 1:**
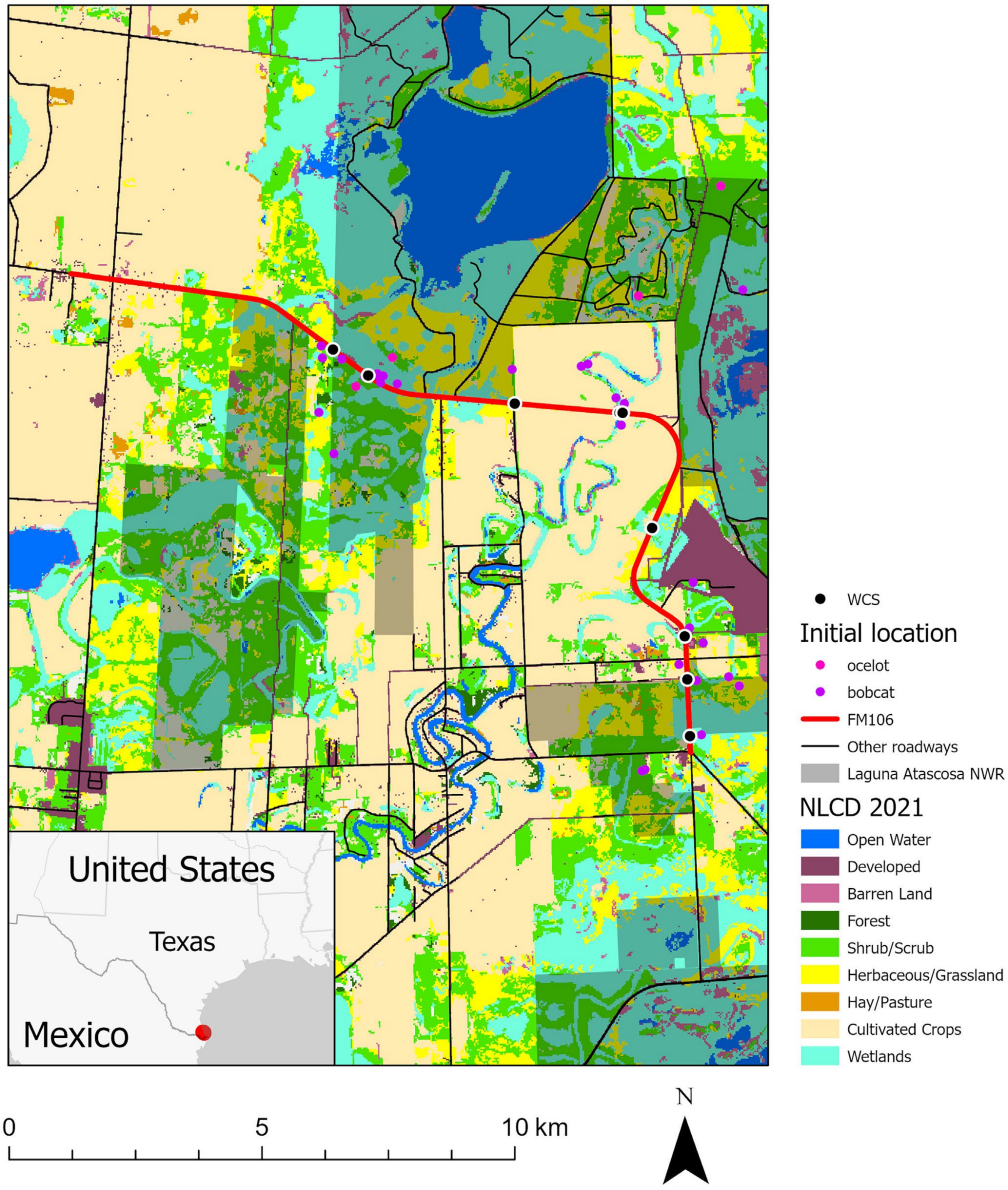
Study area, with location within the United States shown in lower left inset. Initial locations indicate the first location recorded for bobcats ( *Lynx rufus* ) and ocelots ( *Leopardus pardalis* ) marked with GPS collars. The locations of wildlife crossing structures (WCS) are shown along FM106. Land classifications modified from National Landcover Data (2021).

Our study area was the region near LANWR along FM106 (Figure 1). The FM106 Road Improvement project, initiated by TxDOT and the Federal Highway Authority in 2012, aimed to enhance the road by adding shoulders and replacing the old pavement. In 2013, TxDOT and the USFWS amended their Biological Opinion for the project, which, in its updated form, included partial funding for 2 years of monitoring felid use of WCS and outlined a general strategy for this surveillance. The revised conservation measures called for the construction of eight WCS with corresponding fencing, along with the monitoring of felid activities and the use of WCS over time. Construction began in 2015 and was completed in 2019, although individual WCS were completed as early as 2017 (Table 1). The average daily traffic volume on FM106 increased by approximately 40% from 969 mean vehicles/day in 2014 to 1367 mean vehicles/day in 2021, and the posted speed limit increased during the same period following road improvements (80.5 to 96.6 kph [Texas Department of Transportation (TxDOT) 2022]). Before construction, poor road conditions were observed to have restricted motorists' speed to levels below the posted speed limit (H. Swarts, personal observation).

**TABLE 1:** Construction start and end dates for wildlife crossing structures (WCS) installed along Farm‐to‐Market (FM) Road 106 within South Texas.

WCS name	Construction start date	Construction end date
FM1	November 15, 2015	October 15, 2016
FM2	November 15, 2015	September 11, 2017
FM3	November 15, 2015	October 15, 2016
FM4	November 15, 2015	November 15, 2017
FM5	November 15, 2015	November 15, 2017
FMA^a^	NA	NA
FM6	February 15, 2017	July 15, 2019
FM7	February 15, 2017	April 10, 2019
FM8	February 15, 2017	December 14, 2018

^a^ Culvert was not constructed for wildlife use but was sometimes used by wildlife and was monitored as part of this study.

Ocelot habitat in this area was characterized by dense, thorny bushes and shrubs dominated by granjeno ( *Celtis pallida* ), cruzita (*Chromalaena odoratum*), Berlandier's fiddlewood ( *Citharexylum berlandieri* ), honey mesquite ( *Prosopis glandulosa* ), elbowbush ( *Forestiera angustifolia* ), snake‐eyes ( *Phaulothamnus spinescens* ), and colima ( *Zanthoxylum fagara* ), with lesser amounts of brasil ( *Condalia hookeri* ), lotebush ( *Ziziphus obtusifolia* ), whitebrush ( *Aloysia gratissima* ), and coma (*Sideroxylon celastrina*) (Shindle and Tewes 1998). Besides the main thornscrub stands, some native remnant stands of thornscrub also persisted in the form of vegetated areas along oxbows, drainages, and fencerows (Laack 1991). In addition to thornscrub, native habitat in this region consisted of coastal prairies, salt flats, and estuaries. Ocelot habitat in the study area was concentrated in the canopied habitats (shrub/scrub and forest) on and adjacent to LANWR whereas the area to the west and south of LANWR was dominated by agricultural and low‐to‐medium human development and the areas to north contained large private ranches managed for cattle and native game species (Lombardi et al. 2023, Figure 1). Human development in the area increased from 4.1% cover to 18.4% cover between 1987 and 2016 and by 2050 is projected to increase by 33.4% to 39.2% cover (Lombardi, Perotto‐Baldivieso, and Tewes 2020).

### 2.2 Bobcat and Ocelot Movement Data

From 2013 to 2021, we trapped bobcats and ocelots specifically for this project within 2.4 km of FM106, within areas of dense woody cover believed or documented to be used by ocelots. In addition, ocelots and occasionally bobcats were trapped during the study as part of routine population monitoring at LANWR and some of these individuals moved within the 2.4 km buffer while they were monitored and became part of the sample set (see Figure 1 for initial locations of animals). We trapped animals using single‐door wire box traps (108 × 55 × 40 cm, Bobcat Tomahawk Trap, Tomahawk, Wisconsin) that were baited with rock pigeons ( *Columba livia* ) placed in an adjoining compartment (Sternberg and Swarts 2021). We immobilized animals using a pole syringe with a mixture of ketamine hydrochloride (dosed at 6 mg/kg), dexmedetomidine (dosed at 0.05 mg/kg), and butorphanol (dosed at 0.4 mg/kg; Sternberg and Swarts 2021) and beginning in 2019 only ketamine hydrochloride and dexmedetomidine due to a shift in unrelated trapping objectives. Atipamezole was used to reverse anesthesia (dosed at 0.5 mg/kg; Sternberg and Swarts 2021). Lateral photos of the head and both sides of the animal were taken for later identification from camera trap data. Each captured animal was assessed to ensure it was of adequate size to be collared. Given the weight of the GPS collars, collars could only be deployed on animals with a mass of 6.5 kg or greater (Sikes 2016; Sternberg and Swarts 2021). The weight constraint excluded juvenile bobcats and ocelots from receiving GPS collars. We fitted each animal with a mass of 6.5 kg or greater with a Tellus Ultralight GPS collar (mass of approximately 215 g and no more than 3% of the animal's total body mass; Followit AB, Lindesberg, Sweden), administered a sedative reversal mixture of atipamezole and naltrexone (the latter was not needed/administered beginning in 2019), and released animals 1 to 2 h after the sedative was administered when their faculties, specifically visual tracking and muscle coordination, were restored (Sternberg and Swarts 2021).

### 2.3 Camera Traps at WCS

**FIGURE 2:**
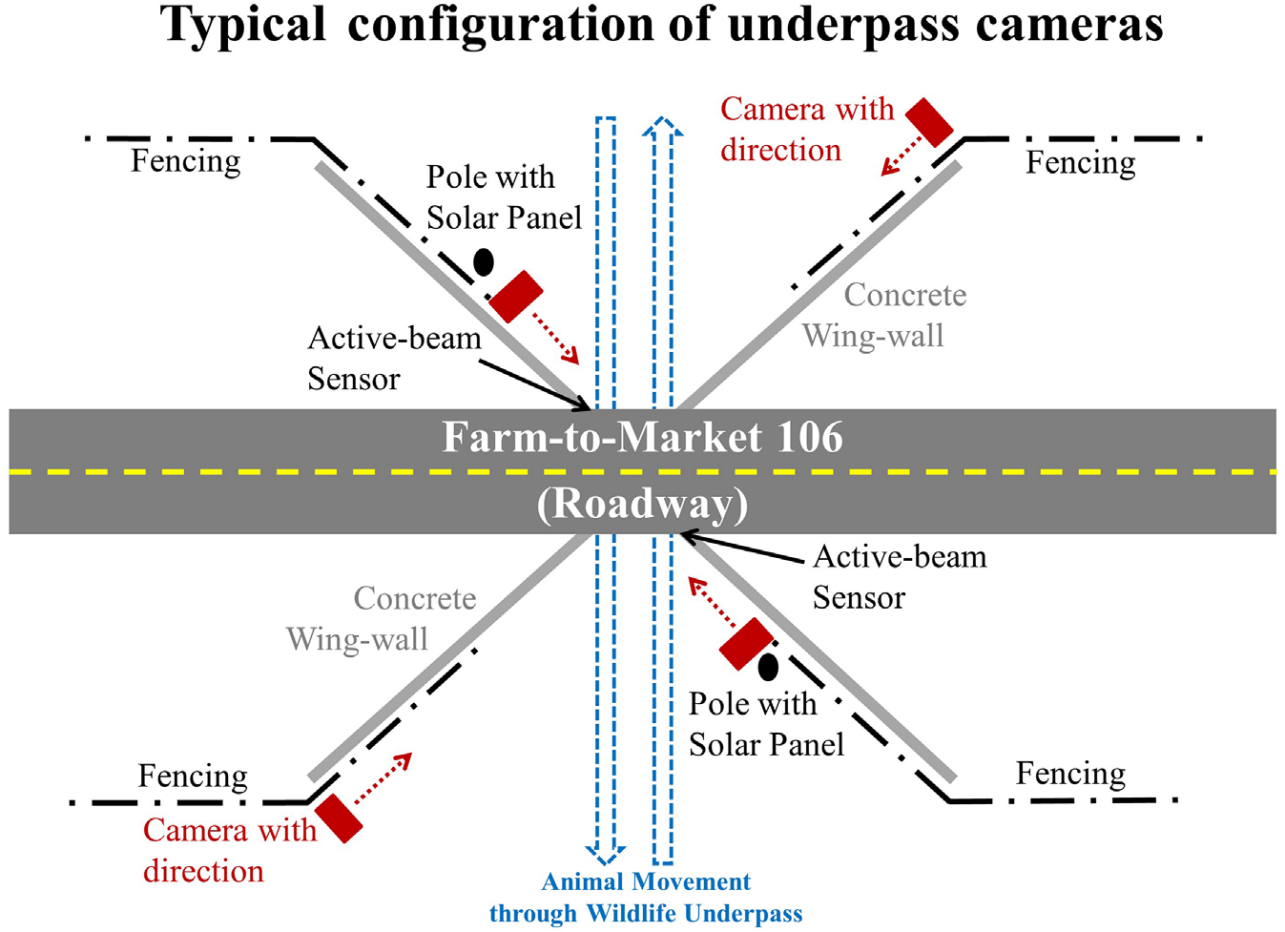
Configuration of cameras used to monitor wildlife crossing structures on Farm‐to‐Market Road 106 (FM106) in Cameron County, Texas during the post‐construction period (figure modified from Sternberg and Swarts 2021).

At each WCS, four cameras were placed to monitor wildlife use of the crossing, with two cameras on each side of FM106 (Figure 2). In the pre‐ and during construction periods, Bushnell Trophy Cam TM, Cuddeback Model C & E, and Cuddeback Model C123 were used; in the post‐construction period, Reconyx HyperFire 2 was used. At each side of FM106 during most of the post‐construction period, the camera closer to the WCS was set up with active infrared using a trip‐beam sensor. This trip beam sensor and its matching reflective pad were set as the entrance to the WCS at the approximate height of a walking bobcat/ocelot (approximately 25 cm, range 20 to 38 cm, to trigger the camera when any movement was detected within the WCS opening; see Sternberg and Swarts (2021) for additional details). Two additional passive infrared cameras were installed, one at each side of the WCS (Figure 2). These cameras were programmed to take three photos per trigger with no quiet period and had sensitivity set to high (Sternberg and Swarts 2021). Post‐construction cameras were initially placed on June 6, 2017, and camera data were analyzed until the last GPS‐collared cat became inactive on April 3, 2021.

### 2.4 Camera Trap Processing

To identify which of the GPS‐collared animals were detected at WCS, we examined only photos taken at the WCS of bobcats and ocelots with GPS collars. Animals were identified using differences among GPS collars and pelage. Collars were initially marked with uniquely patterned duct tape, although the duct tape became compromised with time. We also used individual markings unique to each animal, including markings on inner legs of bobcats (Trolle and Kéry 2003; Heilbrun et al. 2006) for identification. Reference photos taken from when the animal was first captured were used to confirm identification. To be classified as a WCS use event, an animal had to be photographed entering one side of an underpass and exiting the other side (Kintsch et al. 2017; Sternberg and Swarts 2021).

### 2.5 Analysis of FM106 Crossing Rates by Construction Status

We examined the number of on‐roadway crossings by bobcats and ocelots before, during, and after installation of the WCS to assess risk over time. We also examined total crossing rates (all crossings, both using WCS and on‐roadway crossings) to examine whether habitat connectivity across FM106 changed over time with WCS installation. First, we determined the total crossing rate per animal by recording the number of times each animal crossed FM106. To eliminate the possibility that GPS collar error (estimated at 10 m or less) was responsible for erroneously concluding that a crossing occurred when the animal was traveling close to but parallel to the road, we created a buffer that included the road and 10‐m buffer strips outside of each road edge along FM106; a crossing had to include one location on either side of this buffer to be counted as a crossing. To determine which of these crossings used a WCS, for all crossings that occurred after the WCS were installed, we examined photos taken at the WCS to match GPS‐collared animals to the photos taken. Animals were identified according to (1) time and date, (2) GPS collar visible in photo, and (3) visible markings. If there were no photos at a WCS that could be matched to a given crossing event, that event was categorized as an on‐roadway crossing.

We examined the crossing rate (number of crossings of FM106 per day monitored) for both on‐roadway crossings and all crossings over time in a general linear mixed‐model framework using a binomial model. The response variable was number of crossing events per number of days the animal was monitored. Period (before, during, and after installation of WCS and associated fencing) was included as a fixed effect. In addition, we included the log of the mean number of locations taken per day as a fixed effect to account for animals with a shorter time interval between sequential locations potentially detecting more crossing events. To account for differences in the area traversed by different animals, we included autocorrelated kernel density estimators (AKDE) for each animal (Fleming and Calabrese 2023). Unlike conventional kernel density estimators, the AKDE does not assume independence among sequential locations and is thus better suited to high‐frequency location data and variations in fix rates among animals (Silva et al. 2022). We estimated AKDE from the locations of each animal using the “ctmm.select” and “akde” functions from the “ctmm” package (Version 1.2.0, Fleming and Calabrese 2023). We also included the distance from the geometric centroid of each animal's locations to the closest segment of FM106 (distance to FM106) and interaction with AKDE size and distance to FM106 to account for differences in crossing rates due to the combination of proximity to FM106 and size of the area traversed. The AKDE and distance to FM106 predictor variables were initially included as quadratic effects to allow more flexibility in the fitted relationships. We compared species as well as species and sex groups (male bobcats, female bobcats, and male ocelots as there were no female ocelots were included in the study). Individual cat ID was included as a random effect with a random intercept to account for some animals being tracked during multiple construction periods. Predictor variables were examined for correlation and only included in the same model if Pearson's correlation was below |0.7|. Continuous predictor variables were examined for normality and log‐transformed if needed. We also examined beta values for significance and removed models that had uninformative parameters (Arnold 2010). All models were compared and evaluated using AIC_c_. We used model averaging if multiple competing models had ΔAIC_c_ < 2; otherwise, we made inferences only to the model with the lowest AIC_c_ value (Anderson and Burnham 2002).

### 2.6 Analysis of the Influence of Traffic Levels and Time of Day on FM106 Crossing Events

To test for the factors that best predicted the type of crossing event (on‐roadway, WCS, none) that occurred during the interval between sequential locations, we fit a multinomial model with crossing type as the response variable. For this analysis, data were restricted to the post‐construction period when WCS use was possible. Traffic level, time of day, sex, and species were included as predictor variables. Traffic count by hour and year between 2013 and 2021 was taken from the TxDOT's Statewide Traffic Analysis and Reporting System (https://www.txdot.gov/data‐maps/traffic‐count‐maps/stars.html). From this dataset, there were two locations within our study area where traffic was measured along FM106. We used the mean value of the traffic count from the two locations. Because traffic count followed daily patterns and was thus confounded with time of day, we modeled traffic count using a linear model with hour of day as the predictor variable (adjusted *R* ^2^ = 0.63) and then used the residual of this prediction as the variable “traffic level.” Traffic level can be thought of as a relative measure of traffic with high values indicating higher‐than‐expected traffic counts for that hour and low values as the reverse. We also included moon illumination metrics as predictor variables from the “moonlit” package (version 0.1.0; Śmielak 2023) to account for behavioral differences by bobcats and ocelots in response to illumination levels (Sergeyev, Lombardi et al. 2023). From the moonlit package, we included the variables “moonlightModel,” “illumination,” and “moonPhase” using the “calculateMoonlightIntensity” function which predicts moonlight intensity on the ground for a given place, time, and elevation. The “moonlightModel” variable predicted moonlight illumination relative to a full moon, “illumination” predicted the combined moon and twilight illumination in lumens/m^2^, and “moonPhase” predicted the percentage of moon face illuminated. We also included illumination as a categorical variable (dark or light conditions). Because illumination followed daily patterns and was thus confounded with time of day, we modeled illumination using a linear model with hour of day and month as the predictor variables (adjusted *R* ^*2*^ = 0.42) and then used the residual of this prediction as the variable “illumination level.” Illumination level can be thought of as a relative measure of illumination with high values indicating brighter than typical conditions. We included the Euclidean distance between the first location of an interval and FM106 to account for the effect of initial proximity to FM106 on crossing likelihood. In addition, we included the log of time elapsed since the last location to account for longer intervals having more opportunity for a crossing event occurring and individual cat ID as a random effect with a random intercept to account for pseudoreplication of observations from the same animal. We fit these models using the “mblogit” function from the “mclogit” R package (Version 0.9.6; Elff 2022). Model selection was the same as described above for crossing rates.

### 2.7 Analysis of the Effect of FM106 Crossings on Animal Speed

We also examined how WCS usage and on‐roadway crossing events impacted the minimum speed traveled between sequential locations. For this analysis, we used only animals with collars programmed to take locations at least once every 3 h. Minimum speed was calculated as the distance between sequential locations divided by the time elapsed. We used a linear model with log of the minimum speed as the response variable, and crossing event, traffic level, and moon illumination variables were included as predictor variables. To account for variations in speed due to activity level, we also included hour of day (included using sine and cosine to model the 24‐h cycle). The distance from FM106 at the first location of the interval was included to account for the effects of proximity to FM106. The log of time elapsed since the last location was included to account for the effect of variable fix rates and individual cat ID was included as a random effect to control for pseudoreplication. We fit these models using the “lme” function from the “nlme” R package (Version 3.1–163; Pinheiro et al. 2023). Model selection was the same as described above for crossing rates and beta values were assessed for significance and models were ranked using AIC_c_. To incorporate possible temporal autocorrelation among sequential observations, we took the best model from above and added a temporal correlation structure using the “corCAR1” function. This model was compared to the same model without temporal correlation using the “anova” function and inferences made to the most supported model.

### 2.8 WSC Use by Bobcats With Inactive Collars

There were seven bobcats during the monitoring period whose collars did not fall off at the end of the battery life of the GPS device. These bobcats were essentially uniquely marked. The GPS units were uniquely marked with duct tape, and pelage markings could be used to distinguish among these seven individuals when duct tape GPS collar markings were not visible or became obscured. Therefore, we tracked WCS use by these bobcats over time to examine if use rates increased as they became acclimated to the presence of WCS.

## 3 Results

### 3.1 Bobcat and Ocelot Movement Data

In total, 35 animals (30 bobcats and 5 ocelots) were fitted with GPS collars between 2013 and 2021 that were either trapped specifically for this study or moved within 2.4 km of FM106 during posttrapping, collar monitoring. All ocelots were male, whereas both male and female bobcats were collared: 13 females and 17 males. The location interval between sequential locations varied over time. Although all the collars deployed specifically for this study were programmed with a maximum 3‐h interval, some animals trapped on LANWR had GPS collars programmed with longer intervals and were included in this study based on their movement within the 2.4‐km buffer around FM106. Most animals had GPS collars that were programmed to record locations at intervals of 3 h or less (*n* = 30), with the most recently deployed collars recording locations every 30 min (*n* = 10).

In the initial dataset, there were 60,571 locations collectively among the 35 GPS‐marked animals (Table S1). Of these locations, 17,341 were recorded preconstruction, 9,279 during construction, and 33,951 post‐construction. The construction status of the WCS refers to the WCS closest to a given animal's location at the time the location was recorded (e.g., an animal could have a “post‐construction” location if the nearest WCS was complete on that date, even if not all WCS were complete).

### 3.2 Crossing Events Made by GPS‐Monitored Animals

All uses of WCS by GPS‐monitored animals, while their collars were active, were during the post‐construction period (37 of 279 total crossing events detected during this period; Table S2). Of the 10 animals monitored primarily during the post‐construction period that were documented crossing FM106, four of them used a WCS at least once. One animal, male bobcat BM351, accounted for 30 of these 37 WCS uses and used WCS to cross FM106 65% of the time (30 of 46 events). One female bobcat (BF340) and one male bobcat (MB335) used WCS to cross FM106 4% of the time (3 of 71 events and 2 of 53 events, respectively) and an additional male bobcat (BM345) used WCS to cross FM106 2% of the time (2 of 108 events).

### 3.3 WSC Use by Animals With Inactive Collars

In addition to documented use of WCS when the GPS collars were active, there were 123 successful uses of WCS documented on camera traps by three bobcats that had GPS collars after the battery on these collars died but when the collar was still attached (Table 2). One female bobcat accounted for 79 of these 123 WCS uses. This bobcat was observed with her kittens in the WCS on two separate occasions. Two of the three animals that retained their GPS collars and that had been documented as having crossed FM106 (i.e., on‐roadway crossing events) while their collars were active were observed later using a WCS. Of the four animals with retained collars that did not cross FM106 while their collars were active, one was later observed using a WCS.

**TABLE 2:** Use of wildlife crossing structures (WCS) by bobcats ( *Lynx rufus* ) with inactive GPS collars.

CatID	WCS uses	WCS daily use rate during duration detected	Period	Year last active	FM106 daily crossing rate when monitored
BF285	0	NA	Pre	2013	0.00
BF296	0	NA	Pre	2015	0.00
BF311	7	0.02	Pre	2015	0.00
BF319	0	NA	During	2016	0.00
BF324	79	0.07	During	2016	0.90
BF340	37	0.04	Post	2019	2.22
BM291	0	NA	Pre	2015	0.02

*Note:* Under CatID, “BF” signifies bobcat female and “BM” signifies bobcat male. WCS uses are the number of documented uses of WCS by that animal, and WCS daily use rate during duration detected is the number of WCS uses divided by the number of days between the first and last recorded WCS use for that animal. Period refers to the status of the closest WCS during most of the animal's locations when the collar was active. The year last active was the most recent year when the GPS collar was active and the FM106 daily crossing rate when monitored refers to the daily crossing rate of FM106 when the animal's GPS collar was active.

### 3.4 Analysis of FM106 Crossing Rates by Construction Status

**FIGURE 3:**
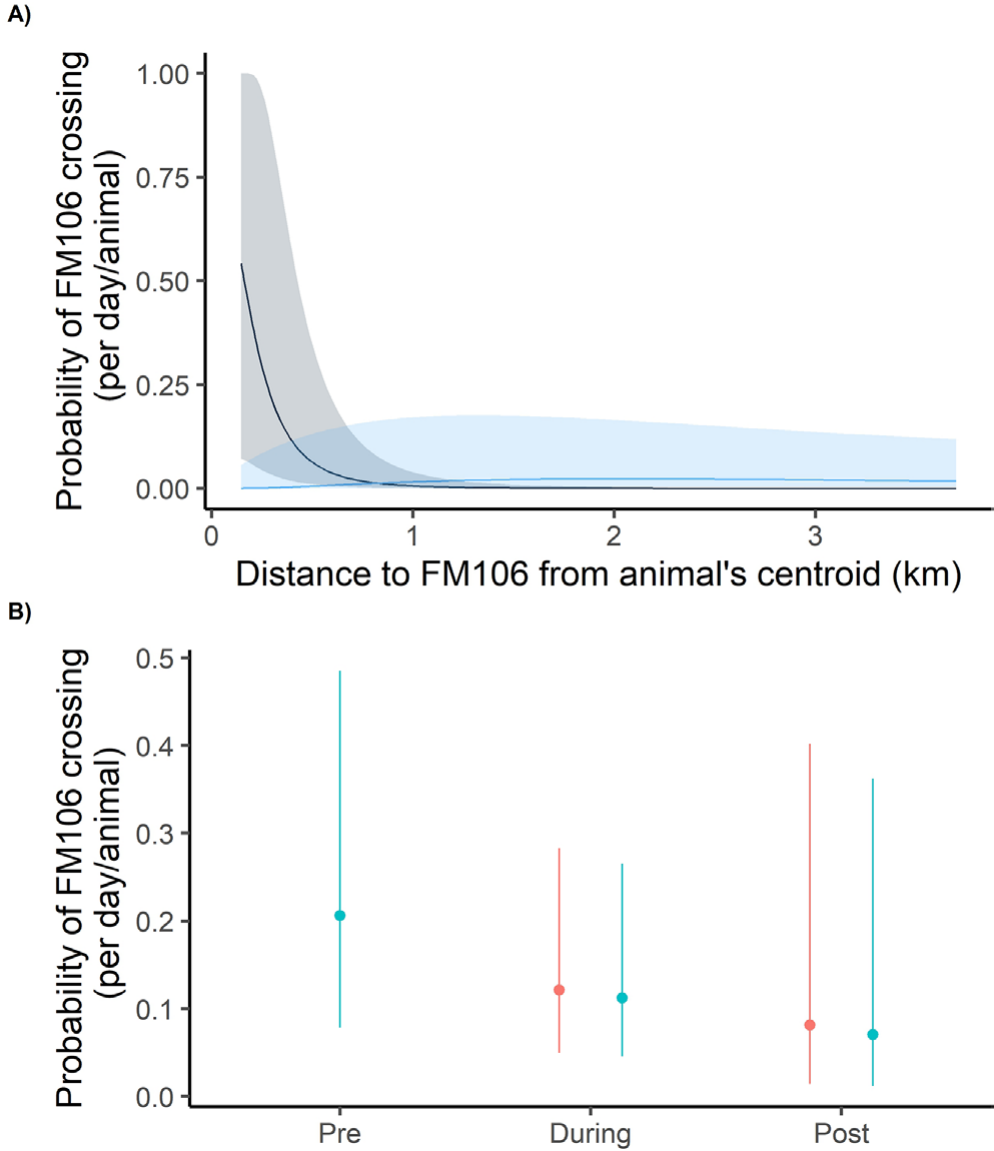
Predicted daily probability of at least one crossing of Farm‐to‐Market Road 106 (FM106) for bobcats ( *Lynx rufus* ) and ocelots ( *Leopardus pardalis* ) in South Texas from 2013 through 2021. (A) Probability of at least one FM106 crossing per day for combined on‐roadway and Wildlife Crossing Structure (WCS) crossings by autocorrelated kernel density estimator (AKDE) size and distance from the centroid of the animal's locations to FM106. The gray line indicates a small AKDE (1.1 km^*2*^), and the blue line represents a large AKDE (24.9 km^*2*^). Shaded areas represent 95% confidence intervals. (B) Probability of at least one FM106 crossing per day by WCS construction status: “pre”: before construction began, “during”: while construction was underway, and “post”: after construction was complete. The predictions were made using the median values for other model variables (autocorrelated kernel density estimator: 12.9 km^*2*^, distance to FM106 1.1 km, and 7.9 locations per day). Blue dots indicate on‐roadway crossings while red dots indicate combined on‐roadway and wildlife crossing structure crossings. Lines represent 95% confidence intervals.

For both the total and on‐roadway crossing analyses, the same model was supported and no other models were competitive. The model contained construction period, the log of the number of locations recorded per day, and quadratic effects for both AKDE size and distance from road along with an interaction effect between AKDE size and distance from roads. Crossing rates were highest for an animal with a small AKDE (112 ha) and a location centroid close to FM106 (Figure 3A). Both on‐roadway and total crossings significantly decreased between the pre‐ and during construction periods (Figure 3B). Neither sex nor species was supported for inclusion in the models. Comparing crossing rates while other predictor values were held at their medians, the animals in this study had a predicted daily probability of at least one on‐roadway crossing of FM106 of 0.21 (95% CI 0.08 to 0.49) during the preconstruction period while this probability dropped to 0.11 (95% CI 0.05 to 0.27) and 0.07 (95% CI 0.01 to 0.36) in the during and post‐construction periods, respectively. Total crossing probabilities including WCS uses were just slightly higher at 0.22 (95% CI 0.08 to 0.51), 0.12 (95% CI 0.05 to 0.28), and 0.08 (95% CI 0.01 to 0.40) for the pre, during, and post‐construction periods, respectively.

### 3.5 Analysis of the Influence of Traffic Levels and Time of Day on FM106 Crossing Events

**FIGURE 4:**
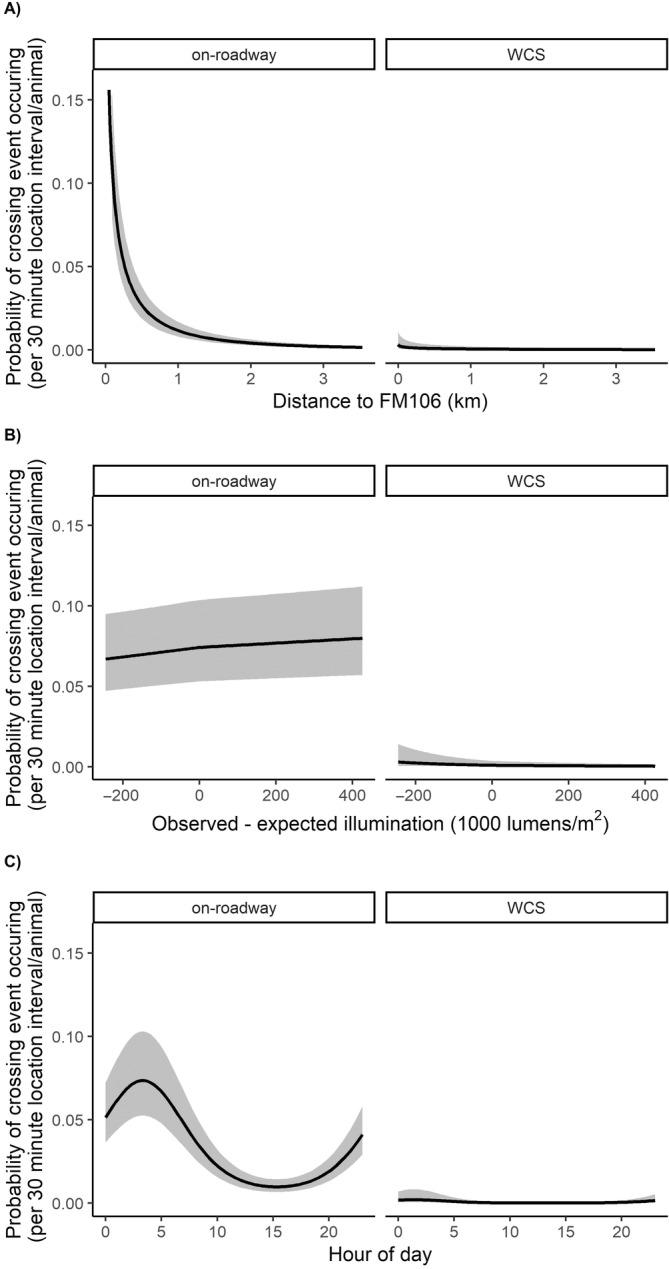
Predicted probability of a crossing event occurring during the post‐construction period between two sequential locations of a GPS‐collared animal by (A) distance to Farm‐to‐Market Road 106 (FM106), (B) observed – expected illumination levels, and (C) hour of day for bobcats ( *Lynx rufus* ) and ocelots ( *Leopardus pardalis* ) in South Texas during 2016 through 2021. Illumination levels represent the difference between observed illumination by moon phase and twilight and that expected according to hour of day and month, for example, negative values indicate darker than typical conditions. Predictions were made using median values for location frequency (locations recorded every 30 min). Shaded areas represent 95% confidence intervals. Crossing type: On‐roadway: road surface crossing of FM106; and wildlife crossing structure: use of a wildlife crossing structure to cross FM106.

The model with the most support in explaining whether and what type of crossing event occurred included traffic level, illumination, distance to FM106, hour of day, and time interval (Table 3). No other models were considered competitive and neither species nor sex was supported for model inclusion. There was some evidence that animals made more on‐roadway crossings during nights with brighter conditions (Figure 4). On‐roadway crossing events were also predicted to be more likely between midnight and the early morning hours (local time; Figure 4). In contrast, WCS use appeared insensitive to illumination and had only weak relationships with hour of day, with more WCS uses predicted outside of midday periods (Figure 4).

**TABLE 3:** Selection results for multinomial models of factors predicting crossing events by bobcats ( *Lynx rufus* ) and ocelots ( *Leopardus pardalis* ) of Farm‐to‐Market Road 106 (FM106) in South Texas, USA, during 2013–2021.

Model	*k*	AIC	ΔAIC	wi
Cosine(2 π hour/24) + sine(2 π hour/24) + distance to FM106^2^ + illumination level + time interval	15	51.96	0.00	0.88
Cosine(2 π hour/24) + sine(2 π hour/24) + traffic level + distance to FM106^2^ + illumination category + time interval	17	5214.85	18.15	0.05
Cosine(2 π hour/24) + sine(2 π hour/24) + traffic level + distance to FM106^2^ + time interval	15	5215.31	18.61	0.05
Cosine(2 π hour/24) + sine(2 π hour/24) + distance to FM106^2^ + time interval	13	5215.64	18.94	0.02
Cosine(2 π hour/24) + sine(2 π hour/24) + traffic level + distance to FM106^2^ + moonlight + time interval	17	5218.54	21.84	0.01

*Note:* The three categories of crossing events were as follows: None, road surface, or wildlife crossing structure use. Traffic levels represent the difference between observed traffic counts and those expected according to hour of day. Distance to FM106 was the Euclidian distance to the closest section of FM106 for the first location in an interval. Time interval was the time elapsed between sequential observations and was included to account for differences due to GPS collar programming. Animal ID was included as a random intercept. We provide the number of parameters (*K*), Akaike's information criterion for small sample size (AIC_c_), the difference in AIC_c_ (ΔAIC_c_), and Akaike weight (w_i_) of the models.

### 3.6 Analysis of the Effect of FM106 Crossings on Animal Speed

**FIGURE 5:**
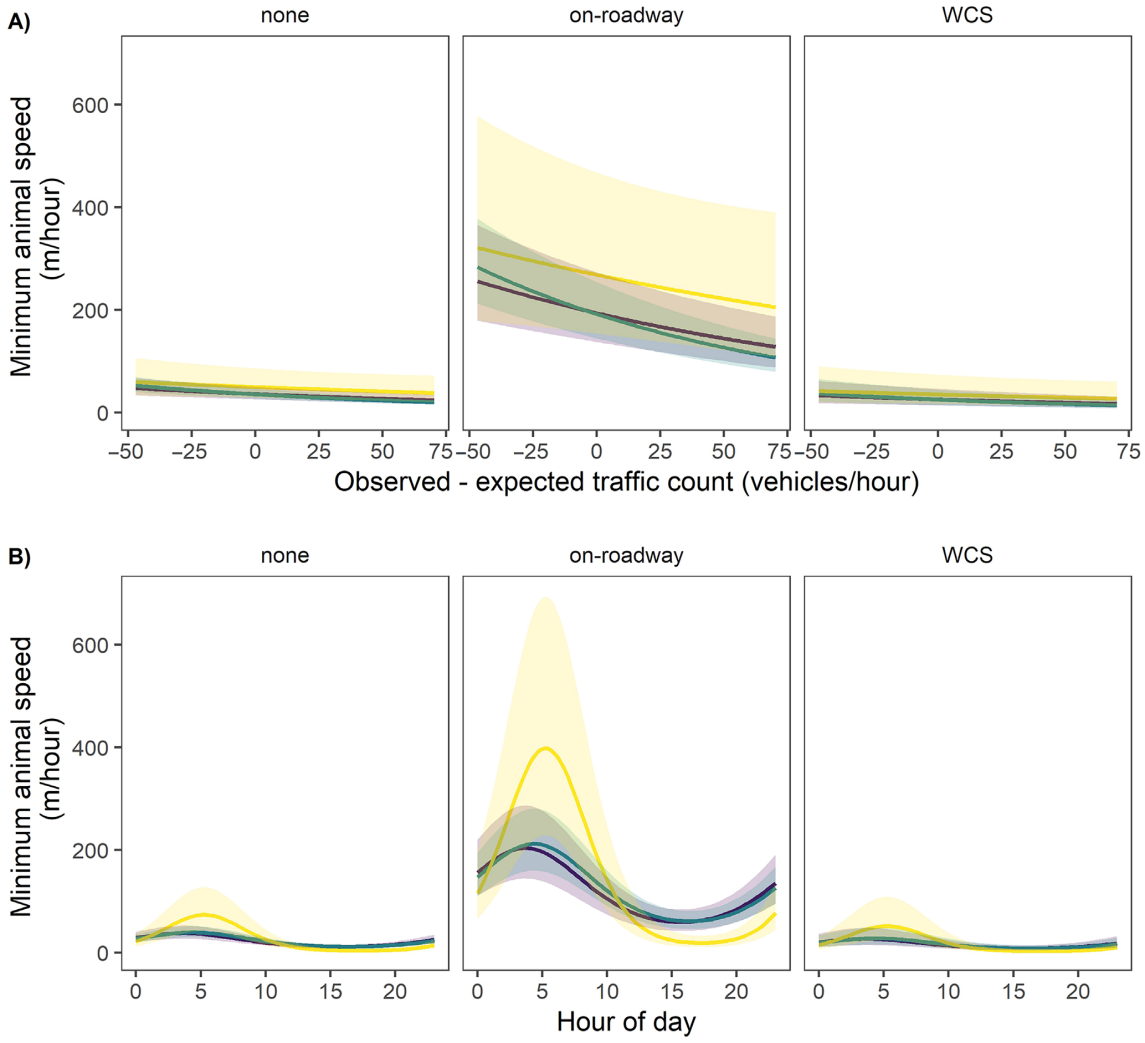
Predicted minimum speed, distance between sequential locations divided by the time elapsed, for bobcats ( *Lynx rufus* ) and ocelots ( *Leopardus pardalis* ) in South Texas from 2013 through 2021 by (A) traffic level and (B) hour of day. Yellow lines represent male ocelots, green lines male bobcats, and purple lines female bobcats. All predictions assume locations were recorded during March, the modal month, that the first location was recorded at the median distance (1.1 km) from Farm‐to‐Market Road 106 (FM106), and, for (A) that the time was 04:00 a.m., when animals were likely to be active. Traffic levels represent the difference between observed traffic counts and those expected according to hour of day, for example, negative values indicate lower than typical traffic counts. Shaded areas represent 95% confidence intervals. Crossing type: none: no crossing of FM106; on‐roadway: road surface crossing of FM106; and WCS use: documented use of wildlife crossing structure to cross FM106.

In the analysis of factors that impacted the minimum speed at which animals moved between sequential locations, the best model included terms for crossing event type, hour of day with interaction with month of year, species and sex, traffic level, distance to FM106 for the first location in an interval, and illumination (Table 4). We found that, while controlling for other variables, animals moved at higher speeds when making on‐roadway crossing events and ocelot males moved faster than bobcats (Figure 5). In addition, bobcat males moved slightly faster than bobcat females. Bobcats moved slower when making on‐roadway crossings when traffic counts were unusually high for that time of day (Figure 5A). Ocelots exhibited a stronger relationship between speed and hour of day than bobcats, with faster movement during early morning hours and little movement during mid‐day (Figure 5B). Speed during WCS usage did not differ significantly from that of noncrossing events for either species (Figure 5).

**TABLE 4:** Selection results for linear mixed models of factors impacting the minimum speed (Euclidean distance between sequential locations/time elapsed) at which bobcats ( *Lynx rufus* ) and ocelots ( *Leopardus pardalis* ) moved.

Model	*K*	AIC	ΔAIC	wi
Crossing + cosine(2 π hour/24) × species/sex + cosine(2 π hour/24) × month + sine(2 π hour/24) × species/sex + sine(2 π hour/24) × month + distance to FM106 x species/sex + traffic level × species/sex + time interval	54	195,257	0.00	1.00
Crossing + cosine(2 π hour/24) × species/sex + cosine(2 π hour/24) × month + sine(2 π hour/24) × species/sex + sine(2 π hour/24) × month + distance to FM106 + traffic level + time interval	50	195,294	36.97	0.00
Crossing + cosine(2 π hour/24) × species/sex + cosine(2 π hour/24) × month + sine(2 π hour/24) × species/sex + sine(2 π hour/24) × month + distance to FM106 + traffic level + illumination category × species/sex + time interval	53	195,351	94.52	0.00
Crossing + cosine(2 π hour/24) × species/sex + cosine(2 π hour/24) × month + sine(2 π hour/24) × species/sex + sine(2 π hour/24) × month + distance to FM106 + traffic level + time interval	50	195,389	132.03	0.00
Crossing + cosine(2 π hour/24) × species/sex + cosine(2 π hour/24) × month + sine(2 π hour/24) × species/sex + sine(2 π hour/24) × month + distance to FM106 + traffic level + moon phase × species/sex + time interval	53	195,389	132.17	0.00
Crossing + cosine(2 π hour/24) × species + cosine(2 π hour/24) × month + sine(2 π hour/24) × species + sine(2 π hour/24) × month + distance to FM106 + traffic level + time interval	47	195,411	154.07	0.00
Crossing + cosine(2 π hour/24) × month + sine(2 π hour/24) × month + distance to FM106 + traffic level × species + time interval	46	195,867	610.29	0.00

*Note:* All data were recorded in South Texas, USA, during 2013–2021. Crossing type indicates if an on‐roadway, wildlife crossing structure use, or no crossing event occurred during a given interval. Traffic levels represent the difference between observed traffic counts and those expected according to hour of day. Distance to FM106 was the Euclidian distance to the closest section of FM106 for the first location in an interval. Time interval was the time elapsed between sequential observations and was included to account for differences due to GPS collar programming. Animal ID was included as a random intercept. We provide the number of parameters (*K*), Akaike's information criterion for small sample size (AIC_*c*_), the difference in AIC_*c*_ (ΔAIC_*c*_), and Akaike weight (w_*i*_) of the models.

## 4 Discussion

Our results suggest that the installation of WCS and associated fencing along FM106 was successful in reducing on‐roadway crossings of FM106 by wild felids. On‐roadway crossings of FM106 for the during and post‐construction phases for both bobcats and ocelots were less than half that of the preconstruction phase. The average daily traffic volume on FM106 increased by 40% over the course of the study (969 mean vehicles/day during 2014 to 1367 mean vehicles/day during 2021 [TxDOT 2022]) and the posted speed limit also increased (80.5 kph to 96.6 kph). In combination with fencing strategically placed adjacent to attractive habitat, the increased speed and traffic volume may have decreased the ability and willingness of animals to make on‐roadway crossings FM106 between 2013 and 2021. However, use of WCS at the end of the study was still low and not sufficient to offset the reduced on‐roadway crossings and subsequent permeability in maintaining habitat connectivity. Carnivores may take years to acclimatize to WCS (Clevenger and Waltho 2000; Seidler, Green, and Beckmann 2018); therefore, a longer period may be needed to evaluate the long‐term effectiveness of these WCS in maintaining connectivity.

Not surprisingly, animals generally crossed FM106 between 10:00 p.m. and 08:00 a.m. local time when these generally nocturnal animals were more likely to be active. Crossings were more likely when the animal was close to FM106 during the first location of an interval. Although we did not find a significant difference between bobcats and ocelots when comparing crossing rates of FM106 after controlling for size of home range and proximity to FM106, the ocelots in our study were located farther from FM106 than the bobcats (Tables S1 and S2). Ocelots have been found to avoid high‐traffic roads in home range placement in South Texas (Veals et al. 2022). This suggests that avoidance of major roadways in home range placement rather than differences in movements within home ranges explains the more frequent crossing by bobcats in this study. This also supports the use of the more abundant bobcat as a surrogate for the ocelot (Schmidt, Lewison, and Swarts 2020) when considering the effects of WCS and associated fencing. There was also weak evidence for higher probability of on‐roadway crossings during brighter than typical periods while controlling for hour and month. We did not find evidence that moon phase itself influenced the probability of on‐roadway crossing or WCS use.

Minimum speed (Euclidean distance between sequential locations divided by time elapsed) during the post‐construction period decreased with increasing traffic levels for both bobcats and ocelots, suggesting the animals moved more cautiously during unusually heavy traffic. As expected, ocelots were more consistently nocturnal than bobcats as well (Figure 5B), with little movement during midday periods. This pattern of nocturnal activity by ocelots and more flexible activity by bobcats is consistent with literature on ocelots and bobcats (Rockhill, DePerno, and Powell 2013; Leonard et al. 2020). We found little evidence that minimum speed was influenced by moon phase or illumination levels. This contrasts with a study on ocelot and bobcat response to moon phase in which ocelots took shorter step lengths during full moons (Sergeyev, Lombardi et al. 2023). However, the effect size in the Sergeyev, Lombardi et al. (2023) study was small (mean step length between 30 min locations = 122.83 m during full moons and 131.57 m during new moons). In addition, other studies have found no relationship between ocelot movement and lunar phase (Pratas‐Santiago et al. 2016), suggesting that this effect is likely to be minor and may vary among populations.

Animals making on‐the‐roadway crossings of FM106 moved faster than when using WCS to cross FM106 or when not crossing FM106, even when controlling for other factors such as season and time of day. In addition, the animals in this study moved slower during unusually heavy traffic volumes while controlling for time of day. This suggests that animals correctly perceive on‐roadway crossings of FM106 as potentially dangerous and minimize the time spent in proximity to this roadway by moving quickly when making on‐roadway crossings while also moving more cautiously when traffic volumes were higher. Jacobson et al. (2016) described four possible behavioral responses to increased traffic volumes by wildlife: nonresponders, pausers, speeders, and avoiders. Speeders respond to the increased risk of higher traffic volumes by increasing speed, as their name would suggest. However, at higher traffic volumes speeders may avoid roadways (Jacobson et al. 2016). Jacobson et al. (2016) described bobcats as being a species that may exhibit both speeder and avoider characteristics while Lovallo and Anderson (1996) found bobcat behavior to be consistent with a speeder response. Both ocelots and bobcats in our study behaved consistently with a speeder response.

There was a great deal of variation in WCS use among individual animals in our study (Table S2). Some resident animals may acclimatize to multiple types of crossing structures, whereas others may be more restricted in which types they are willing to use (Klar, Herrmann, and Kramer‐Schadt 2009; Dexter et al. 2017). Resident animals may vary in their responses, with some individuals responding more favorably than others, illustrating the critical need for adequate sample size of tracked individuals during the post‐construction period. The behavior and presence of conspecifics may have also influenced the willingness of an individual animal to use a WCS. Scent marking of WCS by bobcats in this study area has been documented (Hanley 2022) and bobcats are known to avoid areas scent marked by other bobcats (Bailey 1974). In addition, Hanley (2022) found that bobcat transients in this study area were most likely to use WCS with less preferred habitat nearby, whereas resident bobcats were more likely to use WCS adjacent to dense vegetation and concluded that construction of WCS adjacent to a range of canopy densities may be required to maximize use by both residents and transients.

While occurring after this study ended, ocelots have been observed successfully using the WCS along FM106 (Hanley 2022), supporting the conclusions of this study. In our study, only one ocelot (OM331) was monitored in proximity to FM106 during the post‐construction period. This ocelot did not use any WCS while monitored but also only rarely crossed FM106 (six on‐roadway crossings; Table S2), indicating limited opportunity for WCS use. By focusing on individual animal's behavior rather than counts of WCS use or road mortality surveys, we were able to examine the range of responses to WCS. In addition, our methods were not sensitive to changes in felid abundance over time, which can complicate comparisons of the effectiveness of WCS in reducing road mortality events (Schmidt, Lewison, and Swarts 2021).

There are still some major caveats with this study. Wildlife behavior and use of WCS may change following installation as animals often acclimatize over several years (Klar, Herrmann, and Kramer‐Schadt 2009; Clevenger 2011; Gagnon et al. 2022). At the end of our study, many of the WCS were less than 2 years postcompletion. This is likely less time than needed for these species to adapt to using these structures in their environments (Grilo, Smith, and Klar 2015). For instance, Clevenger (2011) found that it took carnivores 4–6 years to adapt to road crossing structures in Banff, Canada.

In our study we found reduced rates of crossings of both on‐roadway crossings and total crossing events of FM106, implying reduced connectivity as well as reduced exposure to road mortality risk. However, other studies have found that direct mortality from roads often has more immediate and detrimental effects on population persistence than does the reduction in movement between patches (Ascensão et al. 2013; Ceia‐Hasse, Navarro, and Pereira 2018). Fences create barriers to wildlife movement, and they can reduce population persistence when not paired with wildlife passages (Rytwinski et al. 2016). Because fencing is expensive, both in construction and maintenance, the length of fencing paired with WCS is often minimized (Huijser et al. 2016), whereas longer fences are more effective at reducing risk (Huijser et al. 2016; Rytwinski et al. 2016). In a meta‐analysis, Rytwinski et al. (2016) found that fences installed with or without crossing structures reduced wildlife–vehicle collisions by 54%, whereas there was no detectable decrease in mortality from wildlife–vehicle collisions when crossing structures were installed without fencing. The combination of fencing and crossing structures had the highest efficacy rate, with an observed 83% reduction in road mortality of large mammals (Rytwinski et al. 2016). In addition to reducing on‐roadway crossing events, fencing has been shown to increase use of WCS. The probability of an approaching animal crossing through a WCS increased from 0.09 to 0.56 once fencing was added in a study conducted in Arizona (Dodd et al. 2007). Another study in South Texas found that wildlife road mortalities per survey day decreased after five WCS and exclusionary fencing were installed (Yamashita et al. 2021). The reduction in on‐roadway crossing events by the felids in this study supports the idea that the installation of WCS combined with fencing can be an effective way to reduce mortality risks with bobcats and ocelots.

## 5 Conclusions

We found that roadway crossing by bobcats and ocelots in South Texas decreased by more than half after the installation of WCS and associated fencing along with the concurrent roadway improvements and increased speed limit. However, the rate of on‐roadway crossings during the post‐construction period was still higher than the WCS use rate for both species. The animals in our study moved more quickly when making on‐roadway crossings compared to WCS crossings and were more likely to make on‐roadway crossings during nondaylight hours. Animals may take years to acclimate to the presence of WCS, suggesting that the use of these structures may increase over time. In addition, additional fencing that extends fence ends well beyond attractive habitat could help further limit high‐risk road encounters by ocelots and bobcats in this region.

## Author Contributions

**Sarah E. Lehnen:** formal analysis (lead), methodology (lead), visualization (lead), writing – original draft (lead). **Mitch A. Sternberg:** conceptualization (lead), data curation (supporting), writing – review and editing (equal). **Hilary M. Swarts:** conceptualization (lead), data curation (supporting), funding acquisition (lead), project administration (lead), writing – review and editing (equal). **John H. Young Jr:** conceptualization (lead), funding acquisition (lead), project administration (lead), writing – review and editing (equal). **Victoria Hanley:** data curation (lead), investigation (lead), writing – review and editing (supporting). **Richard J. Kline:** conceptualization (supporting), data curation (supporting), writing – review and editing (equal).

## Conflicts of Interest

The authors declare no conflicts of interest.

## Supporting information


**Table S1** Summary of location data from GPS‐tagged animals collected between 2013 and 2021 in South Texas along Farm‐to‐Market Road 106. Species (B = bobcat, O = ocelot), sex (F = female, M = male), and start and end dates refer to the first and last day the animal was tracked. Each location was classified based on the status of the closest wildlife crossing structure (pre‐, during, or post‐construction) at the time the location was recorded. Time interval refers to the mean time between sequential locations.
**Table S2** Sample size and number of crossings by animal. Shortest distance to Farm‐to‐Market Road 106 (FM106) from the centroid location of the animals’ locations is provided as a gauge of the animal’s proximity to FM106; autocorrelated kernel density estimator is provided as a metric of space use. WCS (wildlife crossing structure) use indicates documented use of a WCS by camera traps.

## Data Availability

Data and R code are archived here: https://doi.org/10.7944/3E06‐5T80. Because data include rare endangered species and private property, raw location data have been removed.
