# Supplementary material for: Highway Crossing Rates of Wild Felids Before, During, and After Wildlife Crossing Structure Installation
Source: Ecol Evol. 2024 Dec 23;14(12):e70703. doi: 10.1002/ece3.70703 (PMC11666422; doi:10.1002/ece3.70703)
Supplement: Supplementary file 1 — Table S1 Summary of location data from GPS‐tagged animals collected between 2013 and 2021 in South Texas along Farm‐to‐Market Road 106. Species (B = bobcat, O = ocelot), sex (F = female, M = male), and start and end dates refer to the first and last day the animal was tracked. Each location was classified based on the status of the closest wildlife crossing structure (pre‐, during, or post‐construction) at the time the location was recorded. Time interval refers to the mean time between sequential locations. Table S2 Sample size and number of crossings by animal. Shortest distance to Farm‐to‐Market Road 106 (FM106) from the centroid location of the animals’ locations is provided as a gauge of the animal’s proximity to FM106; autocorrelated kernel density estimator is provided as a metric of space use. WCS (wildlife crossing structure) use indicates documented use of a WCS by camera traps. [file ECE3-14-e70703-s001.docx]

Appendix 1 Table S1. Summary of location data from GPS-tagged animals collected between 2013 and 2021 in South Texas along Farm-to-Market Road 106. Species (B = bobcat, O = Ocelot), Sex (F = Female, M = Male), and start- and end-dates refer to the first and last day the animal was tracked. Each location was classified based on the status of the closest wildlife crossing structure (pre-, during, or post-construction) at the time the location was recorded. Time interval refers to the mean time between sequential locations.

| CatID | Species | Sex | Start-date | End-date | Pre-construction locations (*n*) | During construction locations (*n*) | Post-construction locations (*n*) | Time interval (minutes) |
| --- | --- | --- | --- | --- | --- | --- | --- | --- |
| BF285 | B | F | May 26, 2013 | October 7, 2013 | 123 | - | - | 1440 |
| BF293 | B | F | December 19, 2014 | April 17, 2015 | 800 | - | 35* | 180 |
| BF296 | B | F | December 11, 2014 | May 1, 2015 | 661 | - | - | 180 |
| BF310 | B | F | June 3, 2015 | September 23, 2016 | 2,569 | - | - | 180 |
| BF311 | B | F | October 30, 2015 | October 31, 2015 | 7 | - | - | 210 |
| BF312 | B | F | December 8, 2015 | July 21, 2016 | - | 1,320 | - | 180 |
| BF319 | B | F | April 8, 2016 | November 19, 2016 | 1,779 | - | - | 180 |
| BF324 | B | F | November 10, 2016 | December 10, 2016 | - | 33 | 274 | 180 |
| BF325 | B | F | November 23, 2016 | August 14, 2017 | 659 | 475 | - | 180 |
| BF327 | B | F | December 21, 2016 | April 18, 2017 | - | 16 | 909 | 180 |
| BF328 | B | F | January 10, 2017 | August 11, 2017 | - | 1,692 | - | 180 |
| BF338 | B | F | February 13, 2018 | June 2, 2018 | - | 10 | 5,208 | 30 |
| BF340 | B | F | February 15, 2019 | March 19, 2019 | - | - | 1,517 | 30 |
| BM284 | B | M | May 16, 2013 | March 7, 2014 | 1,445 | - | - | 180 |
| BM288 | B | M | January 14, 2014 | May 21, 2014 | 502 | - | - | 420 |
| BM291 | B | M | May 5, 2014 | November 25, 2014 | 500 | - | 63 * | 360 |
| BM294 | B | M | November 25, 2014 | December 17, 2014 | 55 | - | - | 64 |
| BM306 | B | M | May 9, 2015 | December 13, 2015 | 680 | - | - | 180 |
| BM313 | B | M | December 11, 2015 | February 9, 2016 | 466 | - | - | 180 |
| BM317 | B | M | February 3, 2016 | August 25, 2016 | 1,596 | - | - | 180 |
| BM322 | B | M | May 4, 2016 | November 4, 2016 | 1,365 | - | 5* | 180 |
| BM330 | B | M | January 31, 2017 | August 5, 2017 | - | 839 | 635 | 180 |
| BM332 | B | M | May 6, 2017 | January 22, 2018 | - | 2,517 | 40 | 180 |
| BM334 | B | M | November 2, 2017 | February 5, 2018 | - | 120 | 3,924 | 30 |
| BM335 | B | M | January 9, 2018 | March 16, 2018 | - | - | 3,164 | 30 |
| BM336 | B | M | January 26, 2018 | March 30, 2018 | - | - | 2,949 | 30 |
| BM339 | B | M | February 6, 2019 | May 11, 2019 | - | 831 | 3,661 | 30 |
| BM345 | B | M | December 15, 2020 | February 7, 2021 | - | - | 2,538 | 30 |
| BM350 | B | M | February 1, 2021 | March 31, 2021 | - | - | 2,727 | 30 |
| BM351 | B | M | February 3, 2021 | April 2, 2021 | - | - | 2,709 | 30 |
| OM275 | O | M | February 17, 2016 | October 14, 2018 | - | 939 | 587 | 720 |
| OM276 | O | M | April 18, 2013 | November 8, 2013 | 1,167 | - | 8* | 180 |
| OM283 | O | M | January 30, 2015 | May 22, 2015 | 1,798 | - | - | 180 |
| OM301 | O | M | January 30, 2015 | April 10, 2016 | 1,659 | - | 25* | 180 |
| OM331 | O | M | March 2, 2020 | May 4, 2020 | - | - | 2,973 | 30 |

*^*^Closest crossing structure was FMA, the culvert which, while not designed as a WCS, was still sometimes used by animals and that was completed before this study started.*

Appendix 1 Table S2. Sample size and number of crossings by animal. Shortest distance to Farm-to-Market Road 106 (FM106) from the centroid location of the animals’ locations is provided as a gauge of the animal’s proximity to FM106; autocorrelated kernel density estimator is provided as a metric of space use. WCS (wildlife crossing structure) use indicates documented use of a WCS by camera traps.

| CatID | Days active | Period^1^ | Num. locs. | Mean locs/day | WCS uses | Total FM106 crossings^2^ | Distance from FM106 (m) | AKDE area (ha) |
| --- | --- | --- | --- | --- | --- | --- | --- | --- |
| BF285 | 134 | pre | 123 | 0.9 | 0 | 0 | 1,102 | 64 |
| BF293 | 119 | pre | 835 | 7 | 0 | 7 | 2,725 | 18,067 |
| BF296 | 141 | pre | 661 | 4.7 | 0 | 0 | 1,623 | 104 |
| BF310 | 478 | pre | 2,569 | 5.4 | 0 | 425 | 119 | 245 |
| BF311 | 1 | pre | 7 | 7 | 0 | 0 | 204 | NA |
| BF312 | 227 | during | 1,320 | 5.8 | 0 | 82 | 295 | 114 |
| BF319 | 225 | pre | 1,779 | 7.9 | 0 | 0 | 1,220 | 236 |
| BF324 | 30 | post | 307 | 10.2 | 0 | 22 | 379 | 186 |
| BF325 | 142 | pre | 1,134 | 8 | 0 | 182 | 278 | 249 |
| BF327 | 117 | post | 925 | 7.9 | 0 | 0 | 18,391 | 106,075 |
| BF328 | 213 | during | 1,692 | 7.9 | 0 | 0 | 1,194 | 270 |
| BF338 | 109 | post | 5,218 | 47.9 | 0 | 136 | 1,132 | 926 |
| BF340 | 32 | post | 1,517 | 47.4 | 3 | 56 | 595 | 988 |
| BM284 | 295 | pre | 1,445 | 4.9 | 0 | 143 | 1,993 | 10,670 |
| BM288 | 127 | pre | 498 | 3.9 | 0 | 0 | 2,353 | 5,563,516 |
| BM291 | 204 | pre | 563 | 2.8 | 0 | 5 | 1,005 | 3,531 |
| BM294 | 22 | pre | 55 | 2.5 | 0 | 0 | 395 | NA |
| BM306 | 218 | pre | 681 | 3.1 | 0 | 3 | 10,078 | 6,675 |
| BM313 | 59 | pre | 466 | 7.9 | 0 | 0 | 1,106 | 266 |
| BM317 | 203 | pre | 1,596 | 7.9 | 0 | 31 | 1,140 | 311 |
| BM322 | 183 | pre | 1,370 | 7.5 | 0 | 165 | 455 | 258 |
| BM330 | 185 | during | 1,474 | 8 | 0 | 273 | 398 | 1,351 |
| BM332 | 261 | during | 2,557 | 9.8 | 0 | 59 | 4,325 | 24,719 |
| BM334 | 95 | post | 4,044 | 42.6 | 0 | 112 | 247 | 1,448 |
| BM335 | 66 | post | 3,164 | 47.9 | 2 | 51 | 861 | 642 |
| BM336 | 63 | post | 2,949 | 46.8 | 0 | 2 | 1,911 | 390 |
| BM339 | 94 | post | 4,492 | 47.8 | 0 | 122 | 120 | 2,670 |
| BM345 | 53 | post | 2,538 | 47.9 | 2 | 109 | 711 | 1,831 |
| BM350 | 57 | post | 2,727 | 47.8 | 0 | 3 | 985 | 87 |
| BM351 | 58 | post | 2,709 | 46.7 | 30 | 53 | 803 | 1,217 |
| OM275 | 971 | post | 1,526 | 1.6 | 0 | 0 | 3,366 | 1,691 |
| OM276 | 204 | post | 1,175 | 5.8 | 0 | 95 | 2,549 | 32,042 |
| OM283 | 112 | during | 1,798 | 16.1 | 0 | 26 | 1,874 | 26,313 |
| OM301 | 436 | pre | 1,684 | 3.9 | 0 | 0 | 2,805 | 24,092 |
| OM331 | 63 | pre | 2,973 | 47.2 | 0 | 6 | 2,842 | 902 |

*^1^ Denotes the period (pre, during, or post-construction) when the majority (>50%) of locations were recorded. Some animals overlapped multiple periods (see Appendix 1, Table S1).*

*^2^Denotes all crossings of FM106, including those using WCS*
